# Axonal Guidance Using Biofunctionalized Straining Flow Spinning Regenerated Silk Fibroin Fibers as Scaffold

**DOI:** 10.3390/biomimetics8010065

**Published:** 2023-02-04

**Authors:** Cristina Castro-Domínguez, Paloma Lozano-Picazo, Aroa Álvarez-López, Javier Garrote-Junco, Fivos Panetsos, Gustavo V. Guinea, Manuel Elices, Francisco Javier Rojo, Daniel González-Nieto, Luis Colchero, Milagros Ramos, José Pérez-Rigueiro

**Affiliations:** 1 Neurocomputing and Neurorobotics Research Group, Faculty of Biology and Faculty of Optics, Universidad Complutense de Madrid, 28040 Madrid, Spain; 2 Center for Biomedical Technology, Universidad Politécnica de Madrid, Pozuelo de Alarcón, 28223 Madrid, Spain; 3 Departamento de Ciencia de Materiales, ETSI Caminos, Canales y Puertos, Universidad Politécnica de Madrid, 28040 Madrid, Spain; 4 Brain Plasticity Group, Health Research Institute of the Hospital Clínico San Carlos (IdISSC), 28040 Madrid, Spain; 5 Biomedical Research Networking Center in Bioengineering Biomaterials and Nanomedicine (CIBER-BBN), 28029 Madrid, Spain; 6 Biomaterials and Regenerative Medicine Group, Instituto de Investigación Sanitaria del Hospital Clínico San Carlos (IdISSC), 28040 Madrid, Spain; 7 Departamento de Tecnología Fotónica y Bioingeniería, ETSI Telecomunicaciones, Universidad Politécnica de Madrid, 28040 Madrid, Spain

**Keywords:** spinal cord injury, silk fibroin, silk biomaterials, straining flow spinning, axonal guidance, regeneration

## Abstract

After an injury, the limited regenerative capacity of the central nervous system makes the reconnection and functional recovery of the affected nervous tissue almost impossible. To address this problem, biomaterials appear as a promising option for the design of scaffolds that promote and guide this regenerative process. Based on previous seminal works on the ability of regenerated silk fibroin fibers spun through the straining flow spinning (SFS) technique, this study is intended to show that the usage of functionalized SFS fibers allows an enhancement of the guidance ability of the material when compared with the control (nonfunctionalized) fibers. It is shown that the axons of the neurons not only tend to follow the path marked by the fibers, in contrast to the isotropic growth observed on conventional culture plates, but also that this guidance can be further modulated through the biofunctionalization of the material with adhesion peptides. Establishing the guidance ability of these fibers opens the possibility of their use as implants for spinal cord injuries, so that they may represent the core of a therapy that would allow the reconnection of the injured ends of the spinal cord.

## 1. Introduction

Spinal cord injury (SCI) is a disabling condition that leads to the loss of sensory, motor, and autonomic functions [1]. Currently, the World Health Organization (WHO) states that about half a million people suffer SCI each year, of which almost 90% are associated with road traffic accidents, violence, sports, or falls. The severity of this type of injury depends on the height at which it is located in the spinal cord since if it affects the thoracic or cervical level it can cause paraplegia or tetraplegia, respectively [2,3]. For both patients and their families, this poses great physical and psychological problems, as well as a severe economic burden, which can amount to USD 5 million per patient [4].

After an SCI, several processes occur at both the cellular and the molecular levels in the surrounding tissue. The initial acute phase lasts for approximately 72 h after the injury, and it is characterized by the recruitment of microglia, resident astrocytes, and blood neutrophils to the injury site. The astroglial scar is formed in this phase so that the neuroinflammatory spread of the injury is limited. The scar is characterized by a central area made up of activated microglia and macrophages, surrounded by reactive resident astrocytes in the penumbra of the lesion, so that a barrier is formed. At the molecular level, a series of events such as ion imbalance, excitotoxicity, oxidative damage, and inflammation also occur. Subsequently, during the subacute phase, in addition to the recruitment of additional macrophages, and B and T lymphocytes, apoptosis, axonal demyelination, and Wallerian degeneration take place. Finally, in the chronic phase, maturation of the astroglial scar occurs [5], creating a physical barrier characterized by the presence of molecules such as chondroitin sulfate proteoglycans (CSPG) or tenascin-C [6] that hamper axonal growth. Therefore, the formation of this barrier, together with the excitotoxic environment and the low intrinsic regenerating capacity of the central nervous system (CNS), makes the functional recovery of nerve tissue after an injury almost impossible [7].

Current treatments are conservative and limited to surgical decompression, hyperthermia, cerebrospinal fluid drainage, or the usage of anti-inflammatory drugs, such as methylprednisolone [8,9,10]. These therapies are mainly aimed at stabilizing the patient and preventing complications, leaving aside neuroregeneration. To date, despite active research and clinical studies, no standard, effective, and accepted treatment has yet been found to restore the tissue function lost after SCI [4]. At this time, there are several tissue engineering strategies at different preclinical and clinical stages, but none of them have been approved yet by the US Food and Drug Administration (FDA) for the treatment of SCI [11].

Since after injury the cellular organization of the tissue is destroyed, functional recovery of the tissue requires not only the extension of the axons, but also the ability to guide their growth to replicate the organization of the native nervous tissue [12]. In this context, anisotropic scaffolds appear as a promising strategy to induce the directional growth of axons [13]. In particular, the possibility of producing aligned yarns of micro- or nanofibers offers the possibility of using materials that may sustain the mechanical loads to which the scaffold is subjected during implantation [14]. The feasibility of this approach is demonstrated, among others, by the possibility of controlling the differentiation of mesenchymal stem cells to Schwann cell-like cells with the usage of nanofiber yarns of poly(p-dioxane) [15].

Among the materials proposed to fabricate the anisotropic scaffold, silk fibroin appears as an outstanding candidate to favor the ordered growth of neuronal axons. Fibroin is extracted from the cocoons of the silkworm *Bombyx mori* and, in addition to having excellent properties in terms of biocompatibility, mechanical properties, and degradation, can be processed to generate scaffolds in different formats, such as hydrogels, films, or fibers [16]. In addition, and taking advantage of the reactive groups present in its structure, such as amines in the side chains of the lysine residues or in the N-termini of the proteins, it is possible to biofunctionalize the material with different bioactive molecules [17]. The usage of biofunctionalized fibers allows exploiting the paradigm of biocompatibility [18], in which it is established that the interaction of specific cell lineages with the biomolecules present at the surface of the material determines the response of the organism to the implant. In this regard, preliminary works showed that the usage of fibroin fibers is adequate for axonal guidance [12].

Here we show that high-performance fibroin fibers spun with the straining flow spinning (SFS) process can not only be used to direct axonal growth in vitro [19,20], but also that this guiding ability is further modulated through their functionalization with adhesion peptides. These results, combined with the biocompatibility exhibited by regenerated silkworm silk fibers, open the possibility of using biofunctionalized SFS fibers as implants for reconnecting the ends of the damaged nerves after spinal cord injuries.

## 2. Materials and Methods

### 2.1. Extraction and Purification of Silk Fibroin (SF)

The production of silk fibroin (SF) used for the manufacture of fibers is described elsewhere [17]. Silkworm cocoons were kindly provided by Professors José Luis Cenis and Salvador D. Aznar-Cervantes of the Instituto Murciano de Investigación y Desarrollo Agrario y Alimentario (IMIDA; Murcia, Spain). Briefly, 5 g of silkworm silk (*Bombyx mori*) cocoons were degummed for 20 min in a 2% Na_2_CO_3_ (Sigma-Aldrich, St. Louis, MO, USA) solution in deionized water. After drying the fibroin overnight, the protein was dissolved in a 9.3 M LiBr (Acros Organics, Geel, Belgium) solution at a concentration of 20% (*w*/*v*) at 60 °C for 4 h. The fibroin solution was dialyzed against distilled water at 4 °C to remove the salts until a conductance of fewer than 10 μS/cm was measured in the water. The resulting solution was centrifuged at 5000 rpm for 20 min at 4 °C twice to remove any impurities that might remain in the solution. In order to produce a spinnable dope, it is necessary to increase the fibroin concentration to 16% (*w*/*v*), so that the solution was reverse-dialyzed against a solution of 10% (*w*/*v*) polyethylene glycol (PEG) (Thermo Fisher Scientific, Waltham, MA, USA) with a molecular weight of 8000 Da in 50 mM Tris (Fisher Scientific, Waltham, MA, USA) base (pH 8.0) and 1 M CaCl_2_ (Acros Organics, Geel, Belgium) for 24 h at 4 °C.

### 2.2. Fabrication of High-Performance Regenerated Silk Fibers through Straining Flow Spinning (SFS)

**Figure 1:**
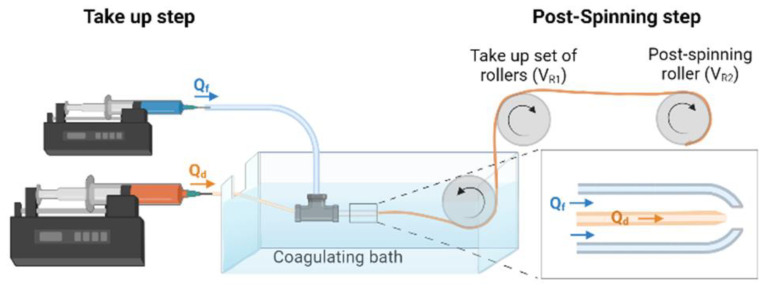
Scheme of a straining flow spinning process with its main elements. The inset shows a detail of the capillary-nozzle system in which the flow of the dope (Q_d_) and of the focusing fluid (Q_f_) are indicated.

A scheme of the straining flow spinning process is presented in Figure 1 and a detailed description may be found elsewhere [19]. Briefly, the dope is injected through a silica capillary (inner diameter 150 μm) inside the space created by a coaxial nozzle, so that the hydrodynamics of the dope jet is controlled by the focusing fluid that flows along the nozzle. The dope jet enters subsequently a coagulating bath, where the solidification of the fiber is completed. The composition of the focusing fluid is the same as that of the coagulating bath and consists of a 1 M solution of acetic acid (Thermo Fisher Scientific) in water/ethanol (20/80 (*v*/*v*)) (Avantor VWR, Radnor, PA, USA). The flow rate of the dope is Q_d_ = 5 μL/min and that of the focusing fluid Q_f_ = 0.5 mL/min. The fibers were collected directly on glass coverslips (Thermo Scientific, Waltham, MA, USA) of 12 mm diameter fixed on a take-up mandrel that rotated at a speed of 15 cm/s. The fibers were retrieved approximately parallel on the coverslips, which were subsequently used in the cell cultures. The fibers were glued by their ends to the coverslip using Dow Corning 3140 RTV Coating biocompatible glue (Dow^®^).

### 2.3. Biofunctionalization of Fibers with Adhesion Peptides through the EDC/NHS Crosslinker Chemistry

N-(3-dimethylaminopropyl)-N′-ethylcarbodiimide hydrochloride (EDC) and *N*-hydroxy succinimide (NHS) crosslinkers were used for peptide labeling and for the immobilization of the peptides to the fibers, as described elsewhere [21] and summarized below.

#### 2.3.1. Labeling of Peptides with FITC

To visualize the immobilization of the peptides on the fibers, the RGD (GRGDSP, Sigma-Aldrich) peptide was labeled with fluorescein 5 (6)-isothiocyanate (FITC, Sigma-Aldrich). Peptides (500 mg/mL) were incubated in a 250 μg/mL FITC solution in PBS at room temperature for 3 h in the dark. To remove the excess FITC molecules, the solutions were dialyzed using Spectra/Por CE dialysis tubes (Cole-Parmer, molecular weight cut-off = 500 Da) against PBS.

#### 2.3.2. Covalent Immobilization of FITC-Labeled Peptide on SFS Fibers

To determine the efficiency of the immobilization protocol, the RGD-FITC-labeled peptide was covalently bound to the SF fibers using the EDC/NHS crosslinking chemistry. For this purpose, the fibers were incubated with the peptides diluted in a 4-morpholino-ethanesulphonic acid (MES, Sigma-Aldrich; 0.3 M, pH = 5.0–6.0) buffer for 1 h. After this time, EDC (Sigma-Aldrich) and NHS (Sigma-Aldrich) crosslinkers diluted in 0.1 M MES were added to a final concentration of 0.125 mg/mL and 0.0315 mg/mL, respectively, and incubated for an additional period of 4 h at room temperature. To remove traces of crosslinkers and unbound peptides, the samples were washed with 10% sodium dodecyl sulfate (SDS, Sigma-Aldrich) in PBS.

The fluorescence of the functionalized fibers was observed with an inverted microscope (Leica DMI 3000B) at the emission wavelength of 520 nm characteristic of FITC. Fluorescence intensity was quantified from the microscope images using the ImageJ software (NIH ImageJ 1.53. https://imagej.nih.gov/ij/ (accessed on 15 May 2022)).

#### 2.3.3. Covalent Immobilization of Peptides on SFS Fibers for Cell Culture Assays

In the case of immobilization on the fibers for cell culture, non-fluorescent RGD and IKVAV peptides were used. The immobilization process was the same as that described in the previous section except for the final cleaning step. In this case, samples were washed twice with PBS for 2 h, and then incubated for 72 h in 0.1 M MES at 4 °C. After this time, the fibers were sterilized with UV irradiation for 20 min and left for 12 h in Dulbecco’s modified Eagle medium (DMEM, Gibco, Waltham, MA, USA) before proceeding with the cell culture.

### 2.4. Cell Lines

Cell adhesion assays were first performed with mesenchymal stem cells (MSCs) obtained from CD1 mouse bone marrow, as described elsewhere [21] and expanded in our laboratory. For the maintenance of these cells, DMEM (Gibco) supplemented with 10% fetal bovine serum (FBS, GE Healthcare Hyclone), 1× penicillin/streptomycin (P/S, Sigma-Aldrich), and 2 mM L-glutamine (Fisher Scientific) was used. Alternatively, human neuroblastoma cells (SH-SY5Y) (ECACC 94030304, Sigma-Aldrich) and cortical neurons obtained from 15-day-old CD1 mouse embryos (E15) were used for the axonal guidance study. All cell cultures were maintained in a humidified atmosphere at 37 °C and 5% CO_2_.

### 2.5. Cell Viability Evaluation Using Fluorescent Live/Dead Cell Assay

MSCs were seeded at a density of 1 × 10^6^ on p35 plates treated for cell culture (BioLite, Thermo Scientific) where both control and RGD- and IKVAV-biofunctionalized fibers had been previously placed. The cells were incubated for 24 h at 37 °C in a humidified atmosphere at 5% CO_2_. After this time, 1 μg/mL Calcein-AM (Fisher Scientific) and 10 μg/mL propidium iodide (PI, Sigma-Aldrich) were added to stain the live cells and incubated for 30 min at 37 °C. Fluorescence was recorded either using a 490 nm excitation filter and a 515 nm emission filter for the number of viable cells (Calcein-AM) or a 535 nm excitation filter and a 617 nm emission filter for the number of dead or late apoptotic cells (PI). Cell viability was measured by assessing the percentage of live cells with respect to the total number of cells. The data were obtained from two independent assays with 3 samples per type of substrate and experiment.

### 2.6. Adhesion Studies

MSC cells were seeded at a concentration of 1 × 10^6^ cells on fibers pre-plated on a p35 plate (Biolite, Thermo Fisher Scientific). For the maintenance of these cells, DMEM supplemented with 10% FBS, 1× P/S, and 2 mM L-glutamine was used. After 2, 4, 6, or 10 h of incubation at 37 °C and in a humidified atmosphere of 5% CO_2_, micrographs of the cells were taken with a bright-field microscope (Leica DMI 3000B). To quantify the total number of cells per sample containing fibers, five representative images (20 × objective magnification) were taken of each sample, and cells were counted using ImageJ.

### 2.7. Axonal Guidance Study with SH-SY5Y

Prior to the guidance study, SH-SY5Y differentiation was induced by using retinoic acid (RA) and brain-derived growth factor (BDNF). For this purpose, the cells were seeded at a density of 3 × 10^4^ cells on p24 well plates treated for cell culture (BioLite, Thermo Scientific) for control, and on coverslips with the fibers with DMEM/F12 medium (Gibco) supplemented with 10% FBS, 1× P/S and 2 mM L-glutamine (d0) (growth medium, GM). After 24 h (d1), this medium was changed to DMEM/F12 medium supplemented with 1% FBS, 1× P/S, 2 mM L-glutamine, and 10 μM all-trans-retinoic acid (RA, Sigma-Aldrich) (differentiation medium 1, DM1). From this moment on, the culture was kept in the dark since the trans-isomer of RA is photosensitive. Five days later (d6), the culture medium was replaced by Neurobasal A medium (Gibco) supplemented with 1× B27 (Gibco), 1× P/S, 2 mM L-glutamine, and 50 ng/mL BDNF (Sigma-Aldrich) (differentiation medium 2, DM2). Subsequent media changes were partially performed to avoid the possible rupture of the growing axons every 3 days. Adhesion and axonal growth were studied over time until day 18, because, once the cells have differentiated into mature neurons, they can only be maintained for up to 2 weeks after terminal differentiation [22].

### 2.8. Axonal Guidance Study Using Murine Primary Cultured Neurons

Cortical neurons were obtained from 15-day-old CD1 mouse embryos, as previously described [23]. Briefly, cerebral cortices were dissociated in 1× Hank’s Balanced Salt Solution (HBSS, Gibco) with 1% P/S. The cortical tissue was enzymatically digested with papain (0.5 mg/mL) (Sigma-Aldrich) for 10 min at 37 °C. Cells were centrifuged at 1500 rpm for 5 min and seeded on 24-well plates treated for cell culture (BioLite, Thermo Scientific) in Neurobasal A culture medium supplemented with 10% FBS, 1% P/S, and 1% L-glutamine. After 24 h, the medium was completely changed to medium supplemented with 1× B27. The medium was partially changed every 2 days. When the cells reached 80% confluence, they were trypsinized and seeded on p24 plates treated for cell culture in the case of controls, and on glass coverslips with nonfunctionalized, RGD-functionalized, and IKVAV-functionalized fibers. Cell adhesion and cell viability were studied after 7 days.

### 2.9. Immunocytochemistry

SH-SY5Y cells were labeled with active cytoskeleton staining using tetramethylrhodamine B isothiocyanate (Phalloidin-TRITC, Sigma-Aldrich). To avoid axon breakage during the washing process, a prefixation process was performed in which paraformaldehyde was added directly to the wells with the cells at a final concentration of 2%. After 2 min, the cells were washed with PBS and fixed for 10 min in a 4% paraformaldehyde solution. Subsequently, they were washed with PBS and permeabilized for 10 min with a 0.1% Triton X-100 (Sigma-Aldrich) solution in PBS. Staining was performed with a 2 μg/mL Phalloidin-TRITC solution in PBS for 60 min.

For mouse cortical neurons, the fixation process was performed in the same manner as previously described. After permeabilization, blocking was carried out using a solution of PBS with 2% BSA (Sigma-Aldrich). Cells were incubated overnight at 4 °C with the primary antibodies anti-glial fibrillar astrocyte protein (GFAP) (1:5000) (Ab7260, Abcam, Cambridge, UK), produced in rabbit, and anti-β-III tubulin (1:1000) (Ab7751, Abcam), produced in mouse, diluted in PBS with 0.1% Triton X-100 and 0.1% BSA. After completing incubation, the samples were washed with PBS three times for 5 min each and incubated with anti-mouse FITC (1:200) (Jackson ImmunoResearch, Cambridge, UK) and anti-rabbit Cy3 (1:800) (Jackson ImmunoResearch) secondary antibodies in PBS with 0.1% Triton X-100 and 0.1% BSA for 3 h at room temperature in the dark. 

Finally, all cells, both SH-SY5Y and cortical neurons, were washed with PBS 3 times for 5 min each and mounted with Fluoroshield with DAPI (Sigma-Aldrich). Samples were observed under a fluorescence microscope (Leica DMI 3000B). In all cases, 5 images were taken on randomly selected points on the well following a Z-pattern.

### 2.10. Description of the Method Employed to Quantify Neural Axon Guidance

To determine the extent of the alignment induced on the growing axons by the presence of the fibers, the guidance factor (ϕ_G_) was defined. This parameter is calculated as the ratio between the number of intersections of the axons and the lines of a grid in the perpendicular direction with respect to the fibers, and the intersections with the lines of the grid directed along the parallel direction. Thus, a factor of one (ϕ_G_ = 1) indicates an isotropic growth, while a value of ϕ_G_ > 1 indicates a preferred alignment of the axons with respect to the fibers.

### 2.11. Statistical Analysis

Statistical analyses were performed with GraphPad Prism 8.0.1. Data are expressed as means ± standard errors of the means (SEM). The Shapiro–Wilk test indicated residuals showed normality. The nonparametric Kruskal–Wallis test was used to analyze the fluorescence results in the case of biofunctionalization of fibers sterilized with different techniques. For MSC viability, adhesion studies, and axonal guidance studies, a two-way ANOVA test was used.

## 3. Results

### 3.1. Biofunctionalization of the Fibers with Fluorescent Peptides

**Figure 2:**
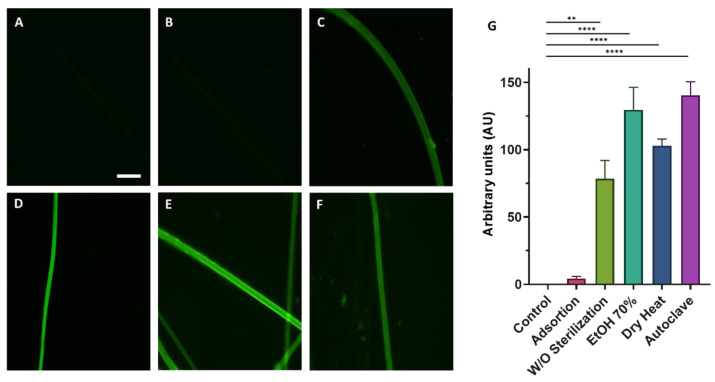
Fluorescence microscopy images of the functionalized fibers using the EDC/NHS crosslinking chemistry with RGD-FITC. Images show the fluorescence of the nonfunctionalized (control) (**A**), fibers incubated with RGD-FITC without crosslinkers (**B**), and fibers functionalized with crosslinkers subjected to different sterilization procedures: no sterilization step (**C**), immersion in EtOH 70% 30 min (**D**), dry heat (2 h at 160 °C) (**E**), and autoclave (**F**) (scale bar = 50 µm). The quantification of the mean fluorescence intensity inferred from the binding of RGD-FITC to SFS fibers after the different sterilization methods is presented as a histogram. The Kruskal–Wallis nonparametric test was performed considering the means ± standard errors of the means and shows significant differences between the functionalized groups and the control (** = *p*-value < 0.01; **** = *p*-value < 0.0001) (**G**).

To determine the efficiency of the biofunctionalization of SFS fibers using EDC/NHS crosslinkers, RGD peptides bound to FITC were used. Since the fibers are intended to be used for cell culture, prior to functionalization they were subjected to different types of sterilization (immersion in Et-OH 70%, autoclave sterilization, and dry heat (160 °C, 2 h) sterilization) to test whether the sterilization step might affect the fiber structure and/or subsequent biofunctionalization process. The unspecific adsorption of FITC to the fibers was also tested by incubating the fibers with the peptides, but without the crosslinkers. Nonfunctionalized fibers were used as a control. Figure 2 shows fluorescence microscopy images of the fibers functionalized with RGD-FITC using different sterilization methods.

For all the sterilization methods, it is observed that fluorescence intensity was higher for the peptide-functionalized samples in the presence of EDC/NHS compared to the control (***p***-value < 0.0001) (Figure 2G). No significant differences were observed between fibers treated with the different sterilization methods. The method chosen to carry out the rest of the experiment was dry heat sterilization due to its simplicity and, in addition, to the absence of leftovers that might be toxic to the cells, for example, EtOH.

**Figure 3:**
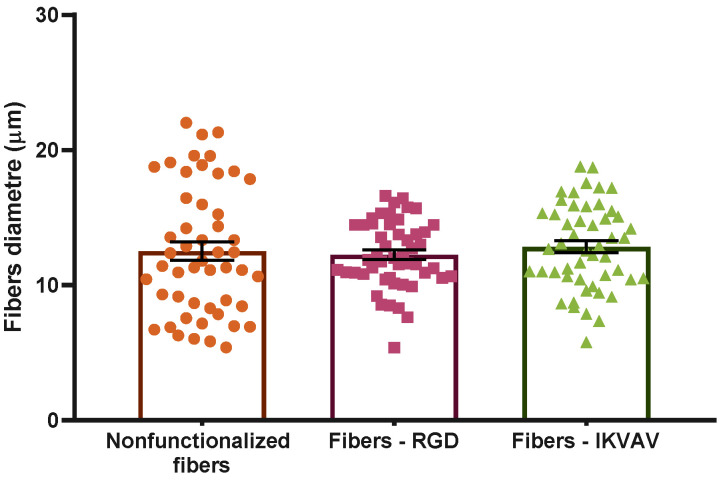
Distribution of the diameters of the different types of fibers. Statistical analysis performed using the Kruskal–Wallis test shows no statistically significant differences between different groups (*p*-value = 0.05). Data are presented as mean ± SEM.

The possible influence of the functionalization of the fibers on their diameter was assessed. The diameter of the control SFS regenerated silk fibroin fibers was 12.5 ± 0.7 µm. RGD- and IKVAV-functionalized fibers yielded diameters of 12.3 ± 0.4 µm and 12.8 ± 0.4 µm, respectively (n = 50). The distribution of the diameters corresponding to each type of fiber is illustrated in Figure 3. As suggested by these data and confirmed by the application of a Kruskal–Wallis test, no significant differences were found in the diameters of the different groups.

In addition to the characterization of the geometrical features of the fibers, previous studies have shown that the mechanical properties of the fibers are not affected by the functionalization process [17].

### 3.2. Evaluation of the Cytotoxicity of Functionalized SFS Fibers on MSC by Fluorescence Live/Dead Cell Assay

**Figure 4:**
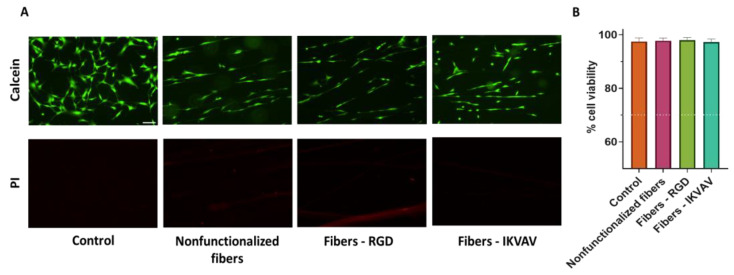
Assessment of viability by Calcein-AM/propidium iodide vital staining. Representative single-channel images of MSC cells at 24 h seeded on polystyrene (control), nonfunctionalized fibers, RGD-functionalized fibers, and IKVAV-functionalized fibers. Live cells are stained green with Calcein-AM, while dead cells are stained red with propidium iodide (PI) (scale bar = 100 µm) (**A**). Quantification of cell viability calculated as the percentage of live cells versus total, determined from five randomly selected images. Data are presented as mean ± SEM (**B**) The dotted line indicates 70% viability, the cut-off value for determining the toxicity of a material.

Previous studies found that titanium samples functionalized with RGD peptides using the EDC/NHS crosslinking chemistry improve the adhesive capacity of MSCs significantly [21]. Consequently, it was checked whether a similar functionalization strategy applied to a different biomaterial, in this case SFS fibers, would affect the MSCs’ survival and adherence. MSCs were seeded on both functionalized and nonfunctionalized SFS fibers, and fluorescence Calcein-AM/propidium iodide staining was used to determine the number of live/dead cells after an incubation of 24 h (Figure 4A). Live cells appear marked in green (Calcein-AM), while dead cells appear in red (PI). To determine the cytotoxicity of the material, cell viability was calculated as the percentage of live cells versus total cells. In this case, as we wanted to test the cytotoxicity of the biomaterial, all labeled cells attached to either the fibers or the substrate were counted for both markers. In order to avoid losing dead cells that might remain floating, the reagents were added directly to the culture medium. Conventionally, a material is considered non-cytotoxic when cell viability is greater than 70% [24]. In all cases in which MSCs were seeded on the biomaterials with the different treatments, high viability was obtained: polystyrene control 98 ± 2%; nonfunctionalized fibers 98 ± 2%; fibers—RGD 98 ± 2%; IKVAV—fibers 97± 3% (Figure 4B). After performing a one-way ANOVA test, no significant differences were found between the groups.

### 3.3. Cell Adhesion Studies (MSCs)

**Figure 5:**
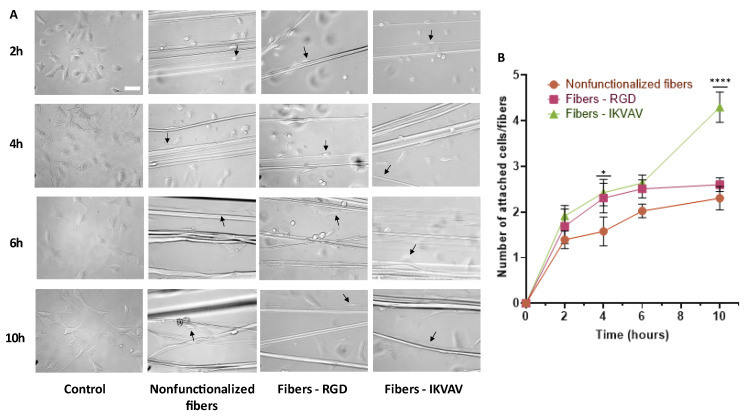
Study of adhesion at 2, 4, 6, and 10 h of the MSCs to the fibers. Bright-field microscopy images of MSCs on polystyrene (control), nonfunctionalized fibers, RGD biofunctionalized fibers, and IKVAV-biofunctionalized fibers (scale bar = 50 µm). Examples of cells attached to fibers are indicated by arrows. (**A**). Statistical analysis of adhesion performed using the two-way ANOVA test shows statistically significant differences at 4 h and 10 h in fibers biofunctionalized with IKVAV compared to control fibers (* = *p*-value < 0.05; **** = *p*-value <0.0001) (**B**).

After seeding 1 × 10^6^ MSCs in each sample, the behavior of MSCs on control and RGD- and IKVAV-biofunctionalized fibers was studied at 2, 4, 6, and 10 h to check the adhesion to the fibers and growth rate of the cells (Figure 5A). The number of cells attached to either control or functionalized fibers was taken as a semiquantitative indication of the adhesion of the MSCs to each type of fiber.

After performing a two-way ANOVA test, statistically significant differences were observed at 4 h (*p*-value < 0.05) for the IKVAV-functionalized fibers compared to the control and at 10 h for the IKVAV-functionalized fibers compared to both the nonfunctionalized and RGD-functionalized fibers (*p*-value < 0.0001) (Figure 5B).

### 3.4. Differentiation of Human Neuroblastoma Cells (SH-SY5Y)

**Figure 6:**
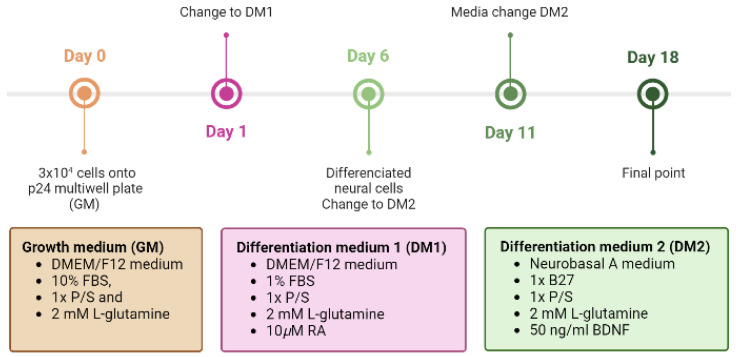
Timetable of differentiation procedure of SH-SY5Y cells to a neuronal phenotype. On the first day of the differentiation protocol (day 0), 3 × 10^4^ cells are plated onto uncoated p24 multi-well plates. After 24 h (d1), the medium is replaced with DM1 containing DMEM/F12 with 1% FBS, 1× P/S, 2 mM L-glutamine, and 10µM RA. On day 6, the medium is changed to DM2 containing Neurobasal A medium with 1× B27, 1× P/S, 2 mM L-glutamine, and 50 ng/mL BDNF.

To study axonal guidance and axon development on the fibers, it is necessary to differentiate SH-SY5Y from a neuronal phenotype. To this end, the cells are subjected to stress environments in the presence of RA and with a low concentration of serum (1% FBS) for 5 days. From this time point onwards, the cells are maintained with a neuron-specific medium supplemented with B27 and BDNF (Figure 6).

**Figure 7:**
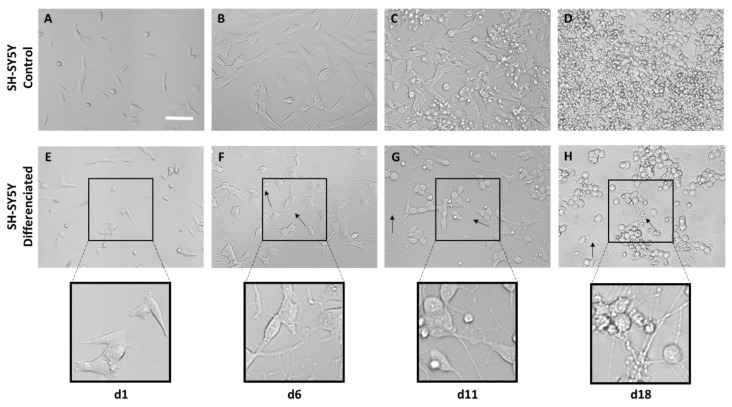
Bright-field microscopy images of the differentiation process of SH-SY5Y cells based on RA and BDNF at 1, 6, 11, and 18 days cultured on polystyrene. Images (**A**–**D**) show the development of control SH-SY5Y cells that have not undergone differentiation. Images (**E**–**H**) show the phenotype change in neurons (scale bar = 50 µm). Axons are marked with arrows.

It can be observed that from 6 d onwards, the cells subjected to the differentiation process begin to develop axons and connections with adjacent cells (Figure 7F). At 11 d and 18 d the presence of more complex neuronal networks can be observed (Figure 7G, H).

### 3.5. Determination of the Axonal Guidance Factor (ϕ_G_)

**Figure 8:**
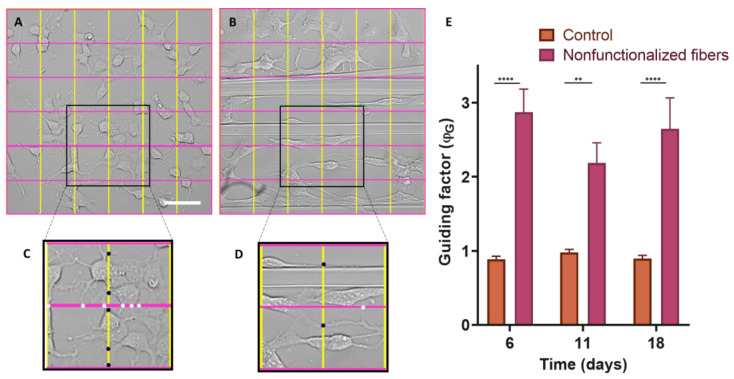
Calculation of the ϕ_G_ guidance factor. Grids divided into 5 equidistant bands both horizontally and vertically were superimposed on bright-field microscopy images of SH-SY5Y cells differentiated for 6 d on control polystyrene (**A**) and on SFS fibers (the grids are oriented parallel and perpendicular to the fibers) (**B**). Two selected areas of the control culture (**C**) and the culture with fibers (**D**) appear enlarged, and the intersection points of the axons with the lines of the grid are marked with black dots (vertical lines perpendicular to the fibers) and white dots (horizontal lines parallel to the fibers). Comparison of axonal guidance factor by two-way ANOVA test considering the means ± standard errors of the means (** = *p*-value < 0.01; **** = *p*-value < 0.0001) (**E**).

In order to determine the efficacy of the guidance factor (ϕ_G_) as a quantitative measurement of axonal guidance, after seeding the SH-SY5Y on control polystyrene plates and SFS fibers, five photos were taken of each well at 6, 11, and 18 days. Statistical analysis using the two-way ANOVA test (Figure 8E) indicates that in the case of control cells seeded on polystyrene, the value of ϕ_G_ is equal to 1, which corresponds to an isotropic growth, resulting in networks without any defined directionality. However, in the case of cells seeded on SFS fibers, it is observed that ϕ_G_ is greater than one, with statistically significant differences at day 6 (*p*-value < 0.0001), day 11 (<0.01), and day 18 (<0.0001). This shows that during the whole process of neuronal differentiation and axonal growth, the cells grow and launch their extensions preferentially in the same direction as the SFS fibers.

### 3.6. Axonal Guiding Study with Human Neuroblastoma Cells (SH-SY5Y)

**Figure 9:**
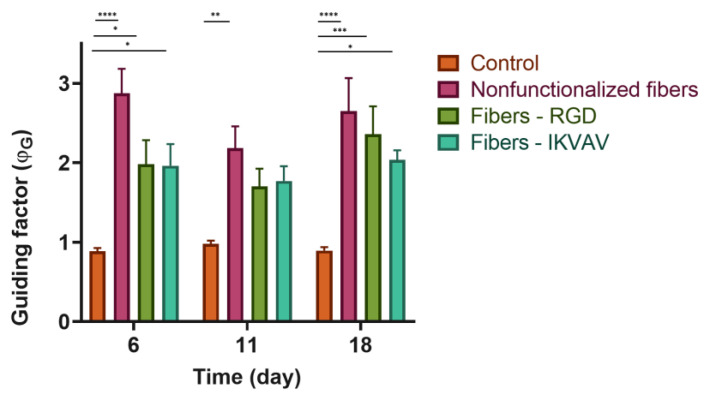
Quantification of ϕ_G_. Guidance factor measured in polystyrene plates, nonfunctionalized fibers, RGD biofunctionalized fibers, and IKVAV-biofunctionalized fibers of differentiated SH-SY5Y at different times 6, 11, and 18 days with a two-way ANOVA test (* = *p*-value < 0.05; ** = *p*-value < 0.01; *** = *p*-value < 0.001; **** = *p*-value < 0.0001).

SH-SY5Y cells were seeded at a density of 3 × 10^4^ on substrates with either nonfunctionalized or biofunctionalized fibers. As a control, cells seeded on polystyrene were also used to determine that the differentiation process did not affect cell viability. The ϕ_G_ values obtained are summarized in Figure 9. At day 6, once the SH-SY5Y are differentiated and have started to form axons, the axons grow following the orientation of the fibers, in contrast with the isotropic growth observed in the control group of cells seeded on polystyrene. In addition, the two-way ANOVA test showed that, in the case of nonfunctionalized fibers, statistically significant differences were observed (*p*-value < 0.0001), while in the case of fibers functionalized with RGD and IKVAV peptides, the differences with respect to the control were somewhat lower (*p*-value < 0.01). At 18 days, the differences in ϕ_G_ remained the same for both nonfunctionalized and functionalized fibers compared to the polystyrene control. This experiment was carried out three times and in each of them three replicates were carried out for each experimental group.

### 3.7. Axonal Guiding Study with Cortical Cells Cultured on SFS Fibers

**Figure 10:**
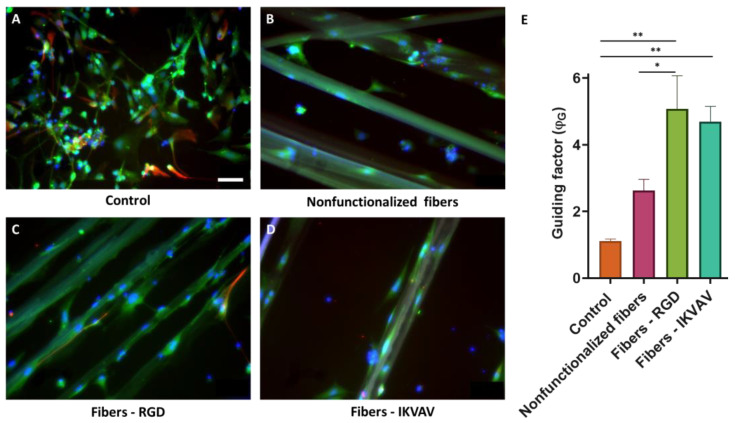
Axonal guidance study with mouse cortical cells at 7 days. Immunofluorescence images of cortical cells seeded on polystyrene (**A**), nonfunctionalized fibers (**B**), RGD-functionalized fibers (**C**), and IKVAV-functionalized fibers (**D**) at 7 days (scale bar = 50 µm). Neurons are labeled in green (**β** III tubulin), astrocytes in red (GFAP), and nuclei in blue (DAPI) (scale bars: 50 μm). Comparison of the ϕ_G_ between control polystyrene, nonfunctionalized fibers, RGD biofunctionalized fibers, and IKVAV-biofunctionalized fibers with cortical neural cells at 7 days with a two-way ANOVA analysis (* = *p*-value < 0.1; ** = *p*-value < 0.01) (**E**).

Mouse cortical cells were cultured on nonfunctionalized and biofunctionalized SFS fibers to determine the orientation of the processes. Immunofluorescence analyses were performed after 7 d to differentiate the main cell types present in the cultures of cortical cells by using β-III-tubulin and GFAP as specific markers for neurons and astrocytes, respectively. Fluorescence microscopy images (Figure 10A–D) showed that the cortical cultures consisted mostly of neurons in all cases. The orientation in which the cells developed was quantified using, as in the previous case, the ϕ_G_ factor (Figure 10E). In the case of cells seeded as a control on polystyrene, a ϕ_G_ close to 1 was observed, which indicates an isotropic axonal growth. In contrast, the values of ϕ_G_ were ϕ_G_ > 1 for the cell cultures in the presence of fibers. These values of ϕ_G_ indicate that the axons are preferably aligned along the direction of the fibers. In addition, and differently from the results obtained with the SH-SY5Y cells, significant differences were identified between nonfunctionalized and functionalized fibers (two-way ANOVA analysis, *p*-value < 0.001). This experiment was carried out three times and in each of them three replicates were carried out for each experimental group.

## 4. Discussion

Spinal cord injury has a negative impact on the life of the patients, as well as on their families and on the society as a whole [25]. Current clinical solutions, such as early spinal cord decompression or methylprednisolone administration, are only contingency measures [26]. This, together with the very limited regenerative capacity of the central nervous system, currently means that the possibility of recovering function after this type of injury is practically null. Although some neurons may survive for a year after injury, the cytotoxic and pro-inflammatory environment created at the injury site, together with glial scar maturation, makes it impossible for them to spread beyond the site of injury [27]. Therefore, one of the main objectives in the field of regenerative medicine and tissue engineering is the creation, with the usage of biomaterials, of implants that can convert the inhibitory environment of the injury into a permissive one to promote and enhance regeneration [28]. Since the extracellular matrix and structure of spinal cord tracts are lost after injury, the ideal scaffolding should provide orientation and support for axon regeneration and prevent disorganized neurite outgrowth [29].

Among the materials proposed for the fabrication of scaffolds, silk fibroin shows a number of advantages compared with other natural or artificial polymers. In this regard, and compared with other natural macromolecules such as collagen, silk fibroin allows producing structures with significantly higher tensile properties. These excellent mechanical properties when the fiber is subjected to tensile loads are of utmost importance for the usage of silk fibroin as axonal guides, since it allows its secure implantation in the organism. In addition, silk fibroin tends to present a reduced number of biological cues compared with other biomolecules, such as hyaluronic acid or collagen, that might induce an undesirable response of the organism to the implant. On the other hand, as is commonly the case when comparing natural and artificial materials in terms of biocompatibility, the former tends to induce a better reaction upon implantation and limit the formation of a connective tissue capsule that tends to appear with the usage of the latter. Consequently, silk fibroin, with its combination of excellent mechanical properties and neurobiocompatibility, appears as a promising material in the field of regenerative medicine and related biomedical applications [16].

The outstanding properties of silk fibroin have been exploited with the development of various spinning processes. Thus, electrospinning of silk fibroin [30], often in combination with other macromolecules, allows the formation of structures that resemble the microstructure of the extracellular matrix (ECM) down to the nanometer scale [31]. Compared with electrospun fibers, SFS fibers exhibit diameters in the range of micrometers, but their excellent tensile properties allow the usage of individual fibers as axonal guides. This work continues in this approach and is intended to demonstrate that biofunctionalized SFS fibers can guide neuronal growth so that the use of these fibers for the fabrication of implants in the case of spinal cord injuries could lead to successful tissue regeneration.

Taking into account that biomaterials interact with their environment through their surfaces and that fibroin lacks cell-binding cues, an attractive strategy to optimize the biointegration of this biomaterial would be its biofunctionalization with extracellular matrix proteins [32]. However, since controlling the conformation of the proteins after binding to the material is a problem, a possible alternative is offered by the use of peptides or short adhesion motifs due to their greater stability and lower steric hindrance. Therefore, in this work, the biofunctionalization of SFS fibers with Arg-Gly-Asp (RGD), a peptide derived from fibronectin, and Ile-Lys-Val-Ala-Val (IKVAV), a peptide derived from laminin, was carried out through the EDC/NHS crosslinking chemistry [33]. The RGD peptide has been used to promote dendritic elongation, adhesion, and proliferation of neural cells in previous works [34,35]. It has also been shown that the peptide IKVAV can not only increase cell viability but also promote neurite outgrowth and neurogenesis [36,37,38].

Fibers were used for cell culturing, so it was necessary to verify that the sterilization of these fibers by different techniques such as dry heat, autoclave, and immersion in EtOH, did not affect the biofunctionalization process. To test the efficiency of this sterilization process, the RGD peptide attached to a fluorescent molecule (FITC) was used. The quantification of the average fluorescence intensity inferred from the binding of RGD-FITC to the SFS fibers when the different sterilization methods are compared shows that the functionalization is not affected by any of these sterilization procedures (Figure 2). Since the simplest form of sterilization of the fibers fixed on the coverslips is dry heat, this was the sterilization method chosen to continue with the subsequent experiments.

Once the efficiency of the peptide immobilization process on the fibers was verified, their possible cytotoxicity was tested. The cleaning process necessary after functionalization is exhaustive and lasts several days since all traces of the crosslinker must be completely removed in order not to affect the survival ability of the cells. A previous work demonstrated that the cleaning protocol used in the present study allows the survival of the cells after the usage of the EDC/NHS crosslinking chemistry to functionalize a material. Less thorough cleaning processes were observed to lead to significant cell death [39]. Given that the functionalization process and the subsequent cleaning of the sample is common to all the experiments that were carried out, MSCs were used to verify the possible toxicity of the process prior to proceeding with other cell lineages. The viability of MSCs growing on nonfunctionalized (control fibers) and RGD- and IKVAV-functionalized fibers was assessed by Calcein and PI staining. Cell viability was around 98% in all conditions (Figure 4), resulting in more than the conventional value of 70%, which is accepted as the threshold value that determines whether a material is biocompatible [24]. These results confirm the good properties in terms of compatibility of the silk fibroin material itself and after its functionalization.

Adhesion studies (Figure 5) confirmed that the adhesion of MSCs on biofunctionalized fibers was improved compared to nonfunctionalized fibers. Specifically, in the fibers functionalized with the IKVAV peptide, statistically significant differences were observed with the rest of the groups at 10 h. An enhancement in the adhesion provided by the immobilization of peptides is thought to translate into a faster adaptation, growth, and phenotypic development of the cells on the fibers, which could favor the release of bioactive molecules by the cells [37,40].

The interaction between neurons and the fibers was assessed using both the SH-SY5Y cell line and mouse embryonic cortical cells. In neuroscience, the use of the SH-SY5Y cell line is extensive, since these cells, in addition to their high reproducibility and viability, can undergo neuronal differentiation by adapting the chemical composition of the cell culture medium. The protocols currently available in the literature are varied, so one of the first steps in the study of axon guidance was the definition and characterization of a method to obtain a phenotype of mature and well-differentiated neurons of these cells in an easy, fast, and efficient manner [41]. In this regard, cells have been incubated with RA and a low serum medium (1% FBS) for 5 days. After this period, the medium was replaced with Neurobasal A, 50 ng/mL BDNF, and 1× B27 (Figure 5). RA, derived from vitamin A, is one of the most widely used agents to induce differentiation. This causes the arrest of cell cycle progression out of G0/G1, increases neurite outgrowth, and upregulates Trk receptors, making cells sensitive to BDNF [42]. While undifferentiated SH-SY5Y cells showed a large and flat cell body with numerous short processes (Figure 7A–D), differentiated cells showed evident extension of long neurites, and the cell body was reduced in size (Figure 7E–H). From day 11, it can be seen how the neuronal population has generated a neural network, apparent from the connection of the neurites with neighboring cells. In addition, it is also worth noting a decrease in cell lysis compared with the controls [22,43]. In the experiments that involve primary cultures, the populations of neurons and astrocytes were identified using the specific expression of β-III tubulin and glial fibrilar astrocyte protein (GFAP), respectively. Thus, it was found that neurons constituted the largest cell population. In addition, it was also observed that astrocytes tended to grow following the direction of the fibers (Figure 10).

To determine the directionality of the axons projected by the neuronal cells, it was necessary to define a quantitative parameter that we refer to as the guidance factor (ϕ_G_), which distinguishes isotropic from directional growth. The guidance factor is calculated by superimposing a grid of vertical and horizontal lines (perpendicular and parallel to the fibers, respectively) and counting the number of intersections between the axons and the lines of the grid. The guidance parameter is defined as the ratio between the intersections along the vertical direction and those along the horizontal direction. A value of the guidance parameter greater than 1 indicates the preferential orientation of the axons along the direction of the fibers.

Control cells on polystyrene plates were shown to grow isotropically over time (ϕ_G_ = 1). In contrast, the cells seeded on the fibers, either the control or functionalized, presented a statistically significant increase in the value of the guidance parameter (Figure 8). This fact implies that the fibers act as physical guides for the growing axons.

Fibers promote axonal guidance in both cell types, SH-SY5Y, and cortical neurons. In the case of the SH-SY5Y cells, it is observed that the guidance parameter is slightly higher for the control fibers than for the functionalized fibers. In contrast, primary cultures from cortical cells reach the highest values of the guidance parameter on biofunctionalized fibers. This result indicates that genuine neurons, obtained from the mouse brain, may be more sensitive to the presence of peptides characteristic of the extracellular matrix, while established cell lines (SH-SY5Y) may have lost some of their original properties and show no differences in the guiding factor between nonfunctionalized and biofunctionalized fibers. In particular, at 7d a significant difference is observed in the fibers functionalized with RGD with respect to the control fibers and IKVAV-functionalized. This might be related to the effect of the latter peptide on neurite outgrowth, in contrast with the RGD peptide, whose function is related to the adhesion of the cells.

## 5. Conclusions

This work demonstrates that it is possible to produce silk fibroin fibers using the SFS technique and functionalize them with desired peptides. The guiding ability of the fibers is quantified by the definition of the guidance factor (ϕ_G_) which distinguishes isotropic from directional growth. The results presented above indicate that the presence of functionalized SFS fibers has an effect on the guidance factor in both types of cells. However, the biofunctionalization carried out with the RGD and IKVAV peptides seems to have a more notable effect only on genuine neurons. In contrast, no such effect is observed in SH-SY5Y cells in which no enhancement in the guiding ability of the fibers is observed between control (nonfunctionalized) and functionalized fibers. Consequently, this work opens the possibility of using functionalized SFS fibers as implants for spinal cord injuries in which the fibers serve as a connection between the injured ends of the spinal cord. The number of possible peptides that are compatible with the biofunctionalization and the possible use of exogenous soluble factors also contribute to the enormous potential of this therapeutic approach.

## Data Availability

The data presented in this study are available on request from the corresponding author. The data are not publicly available due to its large volume.
